# Environmental Impact Assessment of Taza City's Wastewater: Application through Principal Component Analysis

**DOI:** 10.1155/2020/9168569

**Published:** 2020-11-30

**Authors:** Ibrahim Touzani, Mohammed Machkor, Otmane Boudouch, Imane El Machrafi, Rachid Flouchi, Kawtar Fikri-Benbrahim

**Affiliations:** ^1^Laboratory of Microbial Biotechnology and Bioactive Molecules, Sciences and Technologies Faculty, Sidi Mohamed Ben Abdellah University, P.O. Box 2202, Fez, Morocco; ^2^High Institute of Nursing Professions and Health Techniques Annex Taza, Fez, Morocco; ^3^National Electricity and Drinking Water Board, Provincial Laboratory of Taza, P. O. Box 35000, Taza, Morocco; ^4^Environmental & Agro-Industrial Processes Team, Sciences and Technologies Faculty, Sultan Moulay Slimane University, P.O. Box 523, Beni-Mellal, Morocco; ^5^Laboratory of Geosystem-Environment and Sustainable Development, Faculty of Sciences Dhar El Mahraz, Sidi Mohamed Ben Abdellah University, P.O. Box 1796, Fez 30003, Morocco

## Abstract

Taza City is among the Moroccan cities which is in full urban expansion, with a daily wastewater discharge volume estimated at 16534 m^3^/d in 2020, and expected to reach 20056 m^3^/d by 2030. These waters, collected in a combined sewerage network, are directly released into the natural environment without any treatment. Indeed, a large part of this water is discharged into Oued Defali, the main tributary of Oued Larbâa. In order to manage and better understand these discharges impact on the streams crossing this city, wastewater sampling campaigns were carried out for one year from May 2018 to April 2019 at domestic (S1) and industrial (S2) sites. The wastewater physicochemical characterization revealed that these discharges are highly loaded with organic matter in terms of chemical oxygen demand (S1 avg = 1231.44 mg/l and S2 avg = 933.03 mg/l), biochemical oxygen demand (S1 avg = 511.87 mg/l and S2 avg = 464.35 mg/l), and suspended matter (S1 avg = 744.11 mg/l and S2 avg = 578.13 mg/l). The use of principal component analysis (PCA) has allowed us to collect as much information as possible from the database of the physicochemical analyses performed for the studied parameters.

## 1. Introduction

Morocco has subscribed to sustainable development for ten years and has adopted it as its main development choice at the national level, to ensure the management rationalization of natural resources on the one hand and the citizen life's quality continuous improvement on the other hand. Thus, Moroccan Law 10–95 on water imposes the collection and treatment of domestic wastewater in all cities. It specifically requires the establishment of strict standards for urban wastewater treatment based on the receiving environment and discharge standards, as well as steps to prevent the contamination of surface and groundwaters.

Shortage in water resources is increasing worldwide pressure. Indeed, between the years 2010 and 2030, water demand is expected to increase by about 40%, mainly due to agricultural activities [1] and also to the high drinking water consumption due to population growth and urbanization development. Indeed, in 2012, agricultural activities consumed an average of 78% of the world's usable freshwater, 30% in Europe, and more than 85% in sub-Saharan Africa, the Middle East, and North Africa [2]. Moreover, in developed countries, the water quantity consumed is estimated in France, for example, at 150 L/h/d, whereas the world average is only 40 litres [2]. As to Morocco, this consumption is estimated at 85 L/h/d, taking into account the industrial and administrative sectors consumption.

In Morocco, surface and underground water are essential parts of the national hydraulic heritage [3]. However, these resources are faced with quantity and quality problems, linked to global warming and irrational use. Thus, their quality decrease is increasingly aggravated by various polluting discharges, such as urban and industrial wastewater. The annual volumes of urban wastewater discharges have risen sharply in recent decades. They went up from 48 to 506.2 million m^3^/year between 1960 and 2012 and will continue to grow rapidly reaching 741 million m^3^/year by 2030 [4]. However, according to the United Nations Environment Programme [5], the problem of discharging domestic and industrial wastewater into the environment remains common in developing countries, inducing pollution of habitats, water supply resources, and ecosystems [6].

Current environmental problems are manifold and manifest themselves in various territories. In this respect, industrial development and demographic growth of Taza City urban areas lead to a significant increase in terms of liquid discharges volumes and organic mass concentrations produced. Currently, this wastewater is discharged in raw state, with about thirty discharge points into five rivers (oueds) crossing the city, thus contributing to the environmental degradation of these areas and constituting, consequently, a noncompliance with water and environment laws. In this context, the objective of this study is to assess, on the basis of the physicochemical analyses results, the impact of these discharges on the water quality of Oued Defali, which receives a significant part of this wastewater.

## 2. Materials and Methods

### 2.1. Study Site

**Figure 1:**
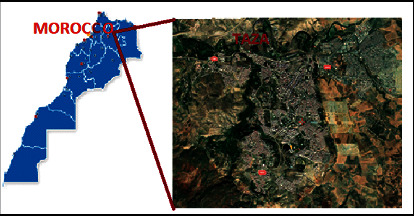
Taza City geographic location (source: Google Earth).

Taza City mentioned in Figure 1 is strategically located on the corridor connecting northeastern Morocco to the country's western agricultural basins. It is built in the valley of Oued Larbâa between the Middle Atlas in the South and the Pre-Rif in the North. The average Lambert coordinates are *X* = 628.230 m and *Y* = 403.162 m. The hydrographic network is composed by Oued Larbâa in the North, Oued El Haddar in the South, and Oueds Defali, Larouireg, and Jaouna in the East. Furthermore, it is characterized by a Mediterranean-type climate: cold and humid in winter and semiarid in summer. Average temperatures vary between 0°C and 38°C (45.4°C absolute maximum). The average rainfall is between 100 and 200 mm/year in the arid zone and exceeds 500 mm/year in the humid zone.

Taza has a unitary sewerage system which covers practically the entire city over a linear distance of 140 km, of which 27 km is the primary network, 17 km is the secondary network, and 96 km is the tertiary network. The sewerage network connection rate is currently estimated at 80%.  Table 1 summarizes the estimates of the flows and pollutant loads of the wastewater in this city.

### 2.2. Sampling

**Figure 2:**
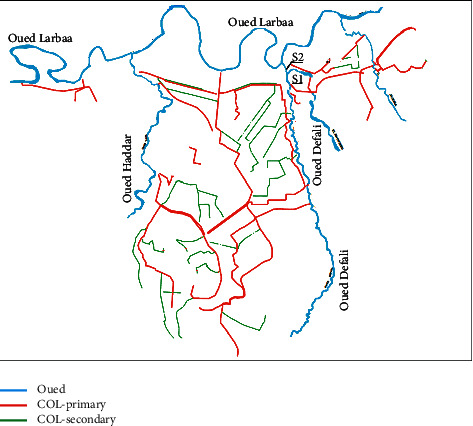
Sites S1 and S2 geographic location.

Sampling campaigns were carried out between May 2018 and April 2019 at two sites: site (S1) is characterized by domestic discharges, and site (S2) is characterized by industrial discharges at a rate of 20 samples per each one (Figure 2).

Samples for physicochemical analyses were taken in one-litre polyethylene bottles in 4℃ temperature to Taza's Provincial Laboratory of the National Office of Electricity and Drinking Water. In situ measurements of temperature and pH were performed using a multiparameter ADWA model AD111. All physicochemical analyses were carried out according to the methods recommended by Rodier [8] (Table 2).

## 3. Results

Detailed results of the studied wastewater physicochemical analyses are shown in Table 3.

## 4. Discussion

### 4.1. Temperature

During our sampling campaigns, the average temperature values of the wastewater from sites S1 and S2 are around 16.10°C and 18.75°C, respectively. The variation in temperature values observed during this study is closely linked to climate change and seasonal periods. Thus, it is very conducive to the development of microorganisms and the phenomenon of wastewater self-purification. However, these recorded results comply with the standards of limit values for direct and indirect discharge into the receiving environment and the quality standards for water intended for irrigation [9, 10]. Moreover, these results are lower than those found in Cotonou City's wastewater in Benin [11] and in Abidjan City's industrial discharges in Ivory Coast [12].

### 4.2. Hydrogen Potential (pH)

The average pH values recorded during our sampling campaigns at sites S1 and S2 are 8.08 and 8.06, respectively. Since these average values are slightly alkaline, these waters may have a detrimental effect on the receiving environment. However, for most aquatic species, the favorable pH range is between 6 and 7.2. However, they are within the Moroccan national standards that set the pH of discharges into the receiving environment at values between 6.5 and 8.5. In addition, the pH values recorded are higher than those found in the wastewater of Oujda City [13] and similar to those found in Kenitra City [14].

### 4.3. Chlorides (Cl^−^)

The average chloride concentrations in the wastewater studied recorded values of 202.72 mg/l at S1 and 181.84 mg/l at S2. These measured values do not exceed the Moroccan standard for water intended for irrigation which is set at 350 mg/l for surface irrigation and 105 mg/l for sprinkler irrigation. This could be explained by the low use of chlorine in cleaning products.

### 4.4. Electrical Conductivity (EC)

The average values recorded for the electrical conductivity in the wastewater at sites S1 and S2 are 1412.42 *μ*s/cm and 1272.24 *μ*s/cm, respectively. In addition, the average conductivity value of the water distributed in Taza City is around 1000 *μ*s/cm. This relatively important mineralization is mainly due to domestic activities and minority industrial activities. However, these values comply with national regulatory standards. They are even lower than those found in the wastewater of Saknia (Kenitra, Morocco) [15].

### 4.5. Suspended Matter (SM) and Turbidity

The concentration of colloidal elements in wastewater gives an idea of the pollution impact in the aquatic environment. The average SM values are in the order of 744.11 mg/l in domestic wastewater and 578.13 mg/l in industrial wastewater. These results are higher than the national standards. Moreover, they are comparable to those found in Kenitra City [14], Sanaa City in Yemen [16], and Azilal City [17]. The effect of SM on the physicochemical characteristics of the water is very harmful, as they can lead to clogging of the soil and reduce the degree of light penetration into the water, which subsequently gives a dirty and cloudy appearance to the waters of Oued Defali receiving these effluents.

Indeed, the turbidity of this wastewater also underwent the same fluctuations as those of the SM contents; it recorded average values of 175.10 NTU in site S1 and 142.31 NTU in site S2. The results obtained show that these waters are highly turbid. Thus, such an increase in turbidity can lead to an increase in the water temperature of the receiving environment.

### 4.6. COD and BOD5

During this study, the mean values recorded for chemical oxygen demand (COD) at sites S1 and S2 were 1231.44 mg/l and 933.03 mg/l, respectively, while those recorded for the biochemical oxygen demand (BOD5) were 511.87 mg/l at S1 and 464.35 mg/l at S2. These values are higher than the national standards for domestic discharge [18] and for direct and indirect discharges. In addition, this wastewater is classified as very poor quality according to the classification of surface water. Indeed, the high concentrations of COD and BOD5 show an excessive consumption of dissolved oxygen, which presents a high load of organic matter discharged into the receiving environment.

### 4.7. Sulfates (SO_4_^2−^)

The average sulphate values recorded during our sampling campaigns are in the order of 111.21 mg/l in S1 and 58.56 mg/l in S2. The concentrations of SO_4_^2−^ in the sites analyzed do not exceed the limit values for the various discharges, whether direct, indirect, or even for water intended for crop irrigation. This is preferable, as sulphates generally generate corrosive hydrogen sulphide, which is toxic and responsible for unpleasant odors.

### 4.8. Nitrate (NO_3_^−^), Nitrite (NO_2_^−^), and Ammonium (NH_4_^+^)

Average nitrate concentrations are 21.17 mg/l at site S1 and 18.57 mg/l at site S2. Nitrates are the most dominant form of nitrogen in water; they generally come from the organic matter decomposition by bacterial oxidation of nitrites. The main effect of nitrates on these waters is their eutrophication, which is triggered when the water is overloaded with nitrates. However, these values remain below the admissible value by Moroccan standards, which are set at 50 mg/l. As a result, the waters studied are not subject to a risk of nitrate pollution. However, these waters are classified as being of average quality according to the surface water quality grid. As for nitrites, the average concentrations recorded in the wastewater discharged are very low, with an average value of 0.32 mg/l in S1 and 0.29 mg/l in S2. The low concentrations of nitrite found in these wastewaters can be explained by the fact that nitrite ion is an intermediate compound, unstable in the presence of oxygen, and easily transformed into nitrate ions. Its concentration is generally lower than that of nitrate and ammonium. Furthermore, this parameter does not constitute a pollution risk for the waters of Oued Defali receiving these effluents. The average ammonium contents are in the order of 11.92 mg/l for site S1 and 7.97 mg/l for site S2. According to the surface water quality grid, the quality of these waters varies from poor to very poor. In general, the presence of ammonium in the water reflects an incomplete degradation of organic matter on the one hand and the presence of urea in domestic wastewater and in urban runoff on the other hand. Ammonium itself can cause a number of problems, such as the development of microorganisms responsible for unpleasant tastes and odors. Similarly, part of the ammonium in alkaline waters can turn into ammonia gas, which is a toxic substance.

### 4.9. Evaluation of the Biodegradability Index COD/BOD5 and BOD5/COD

The combination of the two global pollution parameters COD and BOD5 allows a good approach to biodegradability, with COD representing the organic matter as a whole and BOD5 representing the quantity of organic matter present in the sample that has degraded in 5 days [8]. The COD/BOD5 ratio enables to deduce the water biodegradability. Indeed, it is of great importance in the design of a wastewater treatment system (biological or physicochemical type treatment) and makes it possible to verify whether the wastewater discharged has characteristics of domestic wastewater (COD/BOD5 <3). The mean values of the COD/BOD5 ratio obtained are 2.46 for site S1 and 2.02 for site S2. In comparison with other cities, these results are similar to those found in the wastewater of five cities in Chaouia-Ouardigha Region [19], Sanaa in Yemen [16], Abidjan in Ivory Coast [12], and Azilal [17], M'rirt [20], and Meknes in Morocco [21], but they are lower compared to the values found in the urban commune of Saknia (Kenitra) in Morocco [15] and Mohammedia City [22]. Moreover, the COD/BOD5 ratio of this study is less than 3, which means that this wastewater is rich in organic compounds that are easily biodegradable and for which biological treatment seems quite suitable.

The BOD5/COD ratio is also very useful for assessing the wastewaters biodegradability and origin. The average BOD5/COD ratio values recorded are around 0.41 and 0.49 at sites S1 and S2, respectively. Since these wastewaters have a ratio greater than 0.3, it can be deduced that the substances contained in these effluents are easily biodegradable. Our results are in agreement with those found in the towns of Azilal [17] and M'rirt [20]. However, they are quite different from the value found in the sewage of Mohammedia City [22].

The biodegradability indices are similar for the two study sites S1 and S2; in fact, for most of the cases, the urban wastewater is mainly of domestic origin. It is accepted that the load of organic matter discharged per inhabitant per day (per capita equivalent) varies according to dietary habits, standard of living, habitat type, and population density. Likewise, the unit-type sanitation networks are enriched by rainwater, as in the case of our study, unlike the separate systems which include two distinct pipe networks.

The discharge of urban wastewater (domestic and industrial) without prior treatment can have a considerable environmental impact. Currently, the raw sewage from Taza city is directly discharged into the public water supply system. Faced with this problem, it becomes imperative and urgent to proceed with the technical upgrading of the sanitation sector and to acquire a wastewater treatment plant. Within this framework, several new efficient and low-cost technologies with more technical, economic, and environmental advantages have been investigated for wastewater treatment and reuse, namely, the combination of the Anaerobic Ascending Sludge Bed Reactor (ASBR) technology followed by constructed wetlands either with subsurface flow or free flow [23] or combined Sequential Batch Reactor (SBR) [24]. Another new technology using a combined upflow anaerobic sludge bed followed by an innovative downflow suspended nonwoven fabric for domestic wastewater treatment has been investigated [25]. This process showed a reduction of COD, BOD, and TSS from 349.6, 260.6, and 171.3 to 44, 24, and 27 mg/L, respectively. Another process for the treatment of oil mill wastewater by photovoltaic electrocoagulation uses an external loop aerial transport reactor [26], which is a low operating cost technique giving a COD abatement rate of 79.24%.

## 5. Principal Component Analysis (PCA)

PCA is a statistical technique that can transform the set of quantitative or qualitative data in an individual/variable table into a new set with a small number of variables or major components. PCA was performed using IBM SPSS Statistics 21 software on a data matrix containing 12 physicochemical variables (T, pH, Cl, conductivity, SM, turbidity, COD, BOD5, SO_4_^−^, NO_3_^−^, NO_2_^−^, and NH_4_^+^) of the samples from sites S1 and S2.

Examination of matrix correlation between the variables (Table 4) reveals the presence of one or more variables moderately to highly correlated with one or more other variables: Cond-SO_4_ (0.585), SM-NH_4_ (0.590), COD-SO_4_ (0.594), COD-BOD5 (0.640), Cond-NH_4_ (0.657), BOD5-NH_4_ (0.692), and COD-NH_4_ (0.727).

Thus, the validity of the KMO test average is at 0.684, and the significance of the Bartlett sphericity test is 0.000 (Table 5). This means that not all correlations are equal to zero. Therefore, the variables are dependent on each other.

Based on the results obtained by the PCA on 40 samples with 12 variables, three components were selected because their eigenvalues are greater than or equal to 1 [27].  Table 6 lists the eigenvalues and the total variance explained.

**Figure 3:**
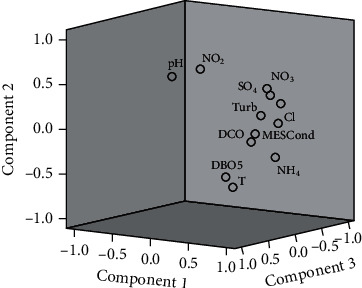
Physicochemical variables on the three-axis plane projection.

The three components explain 64.49% of the variability in the data. The first component accounts for 36.58% of the global inertia via the variables conductivity, MS, turbidity, COD, BOD_5_, SO_4_, NO_3_, and NH_4_. The second component represents 16.20% and is defined by the variables T, pH, and NO_2_, while the third component is explained by Cl with a rate of 11.71% (Figure 3).

## 6. Conclusion

The results of wastewater analyses from Taza City, carried out during this study, showed that the parameters indicative of the organic load had a high level of pollution exceeding the thresholds set by the national standards, which can present a potential risk for the fauna and flora of Oued Defali, for the soils irrigated by these waters and also the populations health. Therefore, this wastewater must not be directly discharged into the watercourses crossing the city, and a treatment suitable for the local climatic and economic conditions must be considered, in order to improve its quality in accordance with the applicable standards and to preserve the city resources.

## Figures and Tables

**Table 1:** Estimates of flows and pollutant loads (Taza City liquid sanitation study [7]).

Year	2020	2025	2030
Water consumption (m^3^/j)	20476	22137	24076
Total average wastewater flow (m^3^/j)	16534	18064	20056
Total pollutant load (kg BOD5/j)	7537	8277	9269

**Table 2:** Physicochemical parameters analysis methods.

Parameter	Analysis methods
Turbidity	Nephelometry
Conductivity	Electrometry
Cl^−^	Titrimétrie
SM	Gravimetry filter method
COD	Colorimetric
BOD5	OXITOP
NO_3_^−^	Molecular spectroscopy method
NO_2_^−^	Molecular spectroscopy method
NH_4_^+^	Molecular spectroscopy method
SO_4_^2−^	Nephelometry

**Table 3:** Physicochemical parameters study sites results.

Parametres	S1			S2		
	Min	Max	Average	Min	Max	Average
Temperature (°C)	7.8	25	16.10	14.4	26.8	18.75
pH	7.78	9.1	8.08	7.72	8.34	8.06
Cl^−^ (mg/l)	180.34	225.42	202.72	113.95	235.4	181.84
Conductivity (*μ*s/cm)	1244.1	1758.9	1412.42	1076.25	1476	1272.24
Turbidity (NTU)	63.44	298.9	175.10	79	210	142.31
SM (mg/l)	267.75	1102.5	744.11	315	945	578.13
COD (mg/l)	847.74	1567.02	1231.44	780	1140	933.03
BOD5 (mg/l)	337.27	672	511.87	338	585	464.35
Sulfates (mg/l)	70	140	111.21	45.51	75.86	58.56
NO_3_^−^ (mg/l)	15.37	25.83	21.17	14.42	24.47	18.57
NO_2_^−^ (mg/l)	0.2	0.45	0.32	0.1	0.5	0.29
NH_4_^+^ (mg/l)	7.05	17.55	11.92	5.35	12.6	7.97

**Table 4:** Correlation matrix.

		T	pH	Cl	Cond	SM	Turb	COD	BOD5	SO_4_	NO_3_	NO_2_	NH_4_
Correlation	T	1.000											
	pH	−0.347	1.000										
	Cl	0.110	−0.218	1.000									
	Cond	0.058	−0.195	0.370	1.000								
	SM	−0.087	−0.050	0.286	**0.510**	1.000							
	Turb	−0.026	0.031	0.240	**0.549**	**0.531**	1.000						
	COD	−0.031	−0.084	0.155	**0.536**	0.430	0.300	1.000					
	BOD5	0.285	−0.061	−0.157	0.404	0.461	0.323	**0.640**	1.000				
	SO_4_	−0.248	−0.026	0.358	**0.585**	0.270	0.346	**0.594**	0.109	1.000			
	NO_3_	0.016	0.169	0.357	0.432	0.265	0.490	0.228	0.049	**0.516**	1.000		
	NO_2_	−0.348	0.304	0.090	0.112	−0.080	−0.014	0.007	−0.094	0.061	0.110	1.000	
	NH_4_	0.285	−0.246	0.329	**0.657**	**0.590**	0.446	**0.727**	**0.692**	**0.541**	0.379	−0.127	1.000

**Table 5:** KMO index and Bartlett test.

Precision measurement of Kaiser–Meyer–Olkin sampling		0.684
Bartlett sphericity test	Approximate chi square	230.535
	Dol	66
	Meaning of Bartlett	0.000

**Table 6:** Total variance explained.

Component	Initial own values			Extraction sum of squares of the factors selected		
	Total	% of variance	% accumulated	Total	% of variance	% accumulated
1	4.389	36.579	36.579	4.389	36.579	36.579
2	1.944	16.203	52.782	1.944	16.203	52.782
3	1.405	11.707	64.489	1.405	11.707	64.489

## Data Availability

All data generated or analyzed during the current study are available from the corresponding author on reasonable request.
